# Opioids and maternal brain–behavior adaptation

**DOI:** 10.1038/s41386-020-00858-7

**Published:** 2020-09-10

**Authors:** James E. Swain, S. Shaun Ho

**Affiliations:** 1 Department of Psychiatry and Behavioral Health, Psychology, and Obstetrics, Gynecology and Reproductive Medicine, Stony Brook University Medical Center, Stony Brook, NY USA; 2 Department of Psychiatry, Psychology and Center for Human Growth and Development, University of Michigan, Ann Arbor, MI USA

**Keywords:** Developmental biology, Biomarkers

Advances in noninvasive brain-imaging of parents with psychological, behavioral, and endocrine measures is pointing to new paradigms for conceptualizing and treating parents with mental illness, including opioid use disorder (OUD) [1]. From 1999 to 2014, the incidence of pregnant women with OUD epidemically quadruped with 2.5% of pregnant women using opioids chronically [2]. Opioid-induced deficits in maternal behaviors have been well-documented in laboratory rodent models [3]. Yet, pregnant women with OUD are commonly treated with medication assisted therapies, including buprenorphine to reduce withdrawal. Buprenorphine is a partial µ-opioid and nociceptin opioid receptor agonist; and also a κ-opioid receptor and δ-opioid receptor antagonist. Animal models of maternal behaviors have found roles for hypothalamus (HYP) in affiliative behaviors and periaqueductal gray (PAG) in aggressive behaviors, which are normally reciprocally modulated [4]. In one rodent model, morphine, a µ-opioid receptor agonists, infused into PAG, disrupted maternal affiliative behaviors, i.e., pup retrieving, without disrupting maternal aggressive behaviors, i.e., predatory hunting, while κ-opioid receptor antagonist infused in PAG increased maternal aggressive behaviors without decreasing maternal affiliative behaviors [5]. Such findings are particularly relevant to the potential effects of buprenorphine given its antagonistic effects on κ-opioid and only partial agonistic effects on µ-opioid receptors. It is thus concerning that buprenorphine replacement treatment may induce more maternal aggressive than affiliative behaviors.

In recent neuroimaging work [2], mothers with buprenorphine replacement treatment showed greater HYP and PAG responses to Own vs. Other’s Baby-Cry as compared to healthy mothers. However, differential functional connectivity in Own vs. Other’s Baby-Cry between the HYP and PAG were associated with parenting stress, suggesting a role of PAG in driving the HYP as a function of parenting stress. These studies support a notion that the buprenorphine may dysregulate the normal balance between maternal caregiving and aggression circuits—each critical for parenting behaviors.

In another neuroimaging study [6], the resting‐state functional connectivity (rs‐FC) between the PAG and HYP was investigated at 1-month (T1) and 4-month postpartum (T2) in mothers treated with buprenorphine replacement treatment (BT) and non‐OUD mothers as a comparison group (CG). Potential bonding impairments were measured using the Postpartum Bonding Questionnaire (PBQ) to explore how rs‐FC with PAG is associated with bonding impairments. Compared to CG, BT mothers differed in PAG‐dependent rs‐FC with the HYP, amygdala, insula, and other brain regions at T1, with many of these differences disappearing at T2, suggesting potential therapeutic effects of continuing buprenorphine treatment. However, the “rejection and pathological anger” subscale of the PBQ at T2 was associated with the T1‐to‐T2 increases in PAG‐dependent rs‐FC with the HYP and amygdala. Thus, maternal bonding problems for mothers with OUD in the early postpartum was linked to connectivity between specific care and defense maternal brain circuits, which may be modulated by buprenorphine treatment.

These studies support potential mechanisms for investigating both benefits and risks of buprenorphine replacement treatment for maternal care and bonding with infants. Furthermore, this framework may be utilized to identify and study new therapeutic targets for mechanism-based treatment strategies for OUD and parent–child bonding disorders.

## Funding and disclosure

This work has been supported by the Research Foundation for the State University of New York (SUNY) and the National Institutes for Health (NIH): National Center for Advanced Translational Sciences (NCATS) via the Michigan Institute for Clinical Health Research UL1TR000433, and National Institute on Drug Abuse (NIDA) R01 DA047336-01. The authors of the manuscript have no conflicts of interest to declare.
